# Desmoplastic small round cell tumor of the kidney: a case report and discussion

**DOI:** 10.3389/fonc.2024.1289773

**Published:** 2024-06-20

**Authors:** Guizhen Huang, Wenqian Huang, Mingxue Liu

**Affiliations:** Department of Pediatric Surgery, The First Affiliated Hospital of Xiamen University, Xiamen University, Xiamen, Fujian, China

**Keywords:** DSRCT, tumor, kidney, surgery, chemotherapy

## Abstract

A 13-year-old boy was admitted to the hospital with 1-month history of neck pain and a 2-week history of bilateral hip joint pain accompanied by low fever. Positron emission tomography-computed tomography (PET-CT) revealed the presence of a malignant tumor in the left kidney with metastases to the left renal hilum, retroperitoneum, para-aortic lymph nodes, and multiple bone sites throughout the body. Given that the patient’s left kidney capsule was intact and the boundary with surrounding tissues was clear, left nephrectomy was performed. Postoperative pathological diagnosis showed desmoplastic small round cell tumor (DSRCT) of the left kidney. CAV-VIP alternating chemotherapy was given 20 days after the first stage surgery. After the end of the 6th cycle, the patient underwent surgery again. The tumor in front of the aorta and postcava, the greater omentum, the retroperitoneal lymph nodes and the hepatic hilum lymph nodes, and the visible tumors in the abdomen were removed. CAV-VIP alternating chemotherapy was continued after the second stage surgery. At the end of the 4th cycle of post operation chemotherapy, radiotherapy was started. An abdominal CT scan conducted 11 months after second-stage surgery did not reveal any recurrence of abdominal tumors; however bone metastases persisted. The patient is currently receiving oral targeted therapy with anlotinib while ongoing follow-up continues.

## Background

Desmoplastic small round cell tumor (DSRCT) is known as an extremely rare and aggressive sarcoma that mainly affects adolescents and young adults. It originates from and primarily affects the peritoneum of the abdomen. The approximately 4:1 male to female ratio is one of most consistent findings in DSRCT (1, 2). Despite progress with multimodality treatments, the results are still poor owing to the high recurrence rate or death within three years for most patients (3). According to the literature published (4), there have been a total of 1,570 reported cases worldwide so far, of which only 16 cases are primary in the kidney. In this paper, we present an unusual case of primary DSRCT that originated in the kidney, and we hope that detailing the characteristics and treatment process of this case can provide useful data for further research on this disease.

## Case report

### Clinical history

**Figure 1:**
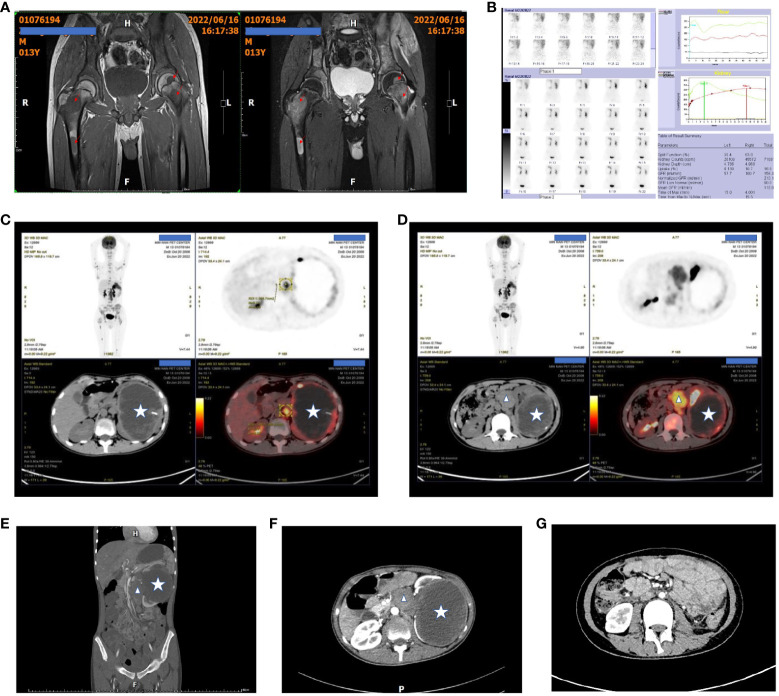
Patient’s pre- and post-treatment imaging data. **(A)** Preoperative hip MRI shows multiple tumors in both femora, with prolonged T1 and T2 signals and high T2-weighted fat-suppressed signals. The arrows indicate the tumor lesions. **(B)** Dynamic renal imaging shows a large, hypovascular mass in the left kidney. **(C, D)** PET-CT scans show a left renal malignant tumor with multiple distant metastases. The star indicates the kidney tumor, and the triangles indicate the metastasis to the inferior vena cava and abdominal aorta. **(E, F)** Preoperative abdominal contrast-enhanced CT scans show the left renal tumor and the metastasis to the inferior vena cava. **(G)** Abdominal contrast-enhanced CT scans at 6 months after the second-stage surgery show no recurrence of intra-abdominal tumors.

A 13-year-old boy was admitted to the hospital with 1-month history of neck pain and a 2-week history of bilateral hip joint pain accompanied by low fever. Oral nonsteroidal anti-inflammatory drugs (NSAIDs) initially provided relief from the symptoms and pain, but the pain recurred readily. The patient was admitted to the rheumatology department before hospitalization due to the suspected diagnosis of rheumatoid arthritis. After admission, treatment for rheumatoid arthritis was ineffective, and the patient experienced significant weight loss. Magnetic Resonance Imaging (MRI) of the hip joint revealed multiple bone abnormalities in the pelvis, spine, and bilateral femurs (**Figure 1A**), raising the suspicion of leukemia or other malignant tumors. PET-CT scans subsequently confirmed the presence of a malignant tumor in the left kidney with multiple systemic metastases (**Figures 1C, D**).

### Physical examination

The patient exhibits significant emaciation, with a body mass index (BMI) of 13.4. Additionally, the patient demonstrates limited range of motion in the cervical and lumbar spine. Both hip joints elicit a positive Fabere test without any signs of erythema or joint swelling. A palpable abdominal mass measuring approximately 15 cm in diameter is present on the left side.

### Preoperative examinations results

Hip joint magnetic resonance imaging (MRI) revealed multiple bone abnormalities in the pelvis, spine, and bilateral femurs, indicating a possible diagnosis of leukemia or other malignant tumors (**Figure 1A**). Renal dynamic imaging exhibited a giant hypovascular mass in the left kidney. The total GFR was 158.4ml/min, the GFR of left kidney was 57.7ml/min, and the GFR of right kidney was 100.7ml/min. (**Figure 1B**). Positron emission tomography-computed tomography (PET-CT) demonstrated the presence of a malignant tumor, possibly left renal cell carcinoma or another type of renal cancer. Additionally, metastases were observed in the left renal hilum, retroperitoneum, and para-aortic lymph nodes as well as multiple bone metastases throughout the body (**Figures 1C, D**). Abdominal contrast-enhanced CT scans show the left renal tumor and the metastasis to the inferior vena cava (**Figures 1E, F**).

### Treatment process

#### First stage surgery

Considering that the patient’s left kidney capsule was intact and the boundary with surrounding tissues was clear, left nephrectomy was performed. During the operation, the capsule vessels of the left kidney were congested, and the size of the left kidney tumor was approximately 18x13x12cm^3^ with an intact renal capsule. The postcaval lymph nodes were found to be swollen and adhered to each other, with a hard texture, about 8x5x3cm^3^, and tightly adhered to the postcava and aorta, making them difficult to separate. Considering that about 800ml of blood had been lost during the operation, the removal of the swollen lymph nodes near the aorta and postcava was abandoned. A total of 4 units of red blood cell suspension and 200ml of fresh frozen plasma were transfused during the operation.

#### Postoperative pathological diagnosis

**Figure 2:**
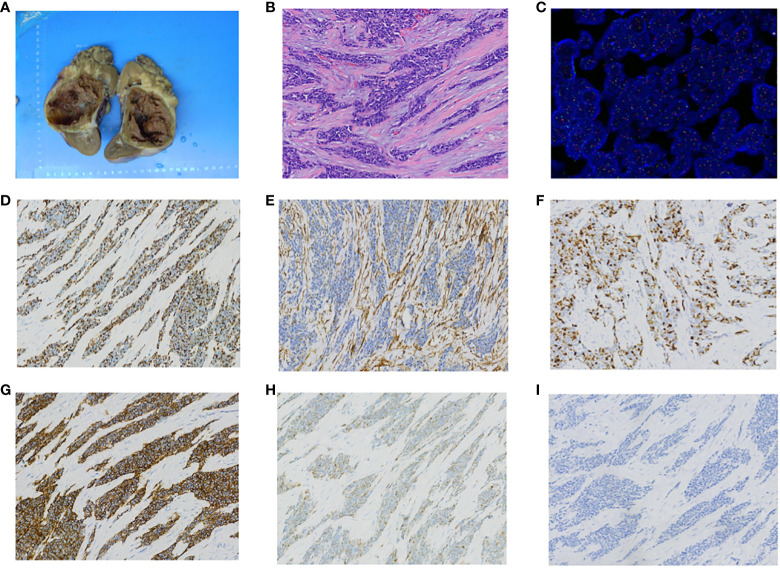
Pathology findings. **(A)** Gross appearance of left kidney tumor. **(B)** HE staining of left kidney tumor shows undifferentiated small round or oval cells with prominent nuclear division. **(C)** FISH testing with a dual-color probe targeting the EWSR1 locus shows approximately 98% of tumor tissue with a red-green-yellow breakage signal, and the percentage of cells with breakage signal is greater than 10%. This suggests the presence of a breakage gene in the affected tissue. **(D)** Desmin is positive on immunohistochemistry staining. **(E)** Vimentin is positive on immunohistochemistry staining. **(F)** Ki67 is positive on immunohistochemistry staining. **(G)** CD56 is positive on immunohistochemistry staining. **(H)** CyclinD1 is positive on immunohistochemistry staining. **(I)** WT-1 is negative on immunohistochemistry staining.

Postoperative pathological examination revealed desmoplastic small round cell tumor infiltrating the surrounding renal tissue of the left kidney, with involvement of the renal capsule and perirenal fat capsule, but no tumor infiltration in the ureter (**Figures 2A, B**). Fluorescence *in situ* hybridization (FISH) analysis demonstrated the presence of EWSR1 breakpoint gene (**Figure 2C**). Immunohistochemistry (IHC) staining showed positive expression for Desmin (**Figure 2D**), Vimentin (**Figure 2E**), Ki67 (**Figure 2F**), CD56 (**Figure 2G**), CyclinD1 (**Figure 2H**), and negative expression for WT-1 (**Figure 2I**). Next-generation sequencing technology confirmed EWSR1-WT1 gene fusion. Microsatellite instability testing did not detect MSI-H. Tumor mutational burden testing indicated a mutation rate of 1.1 mutations per megabase.

#### Postoperative chemotherapy

The CAV-VIP alternating chemotherapy was administered 20 days after the initial stage surgery. The first cycle of chemotherapy consisted of the CAV regimen which comprised cyclophosphamide, pirarubicin and vincristine. The second cycle involved the VIP regimen, including isocyclophosphamide, cisplatin, and etoposide (**Table 1**). The primary complication of chemotherapy is myelosuppression, characterized by leukopenia and thrombocytopenia. The patient eventually developed tolerance to chemotherapy through platelet transfusion and administration of hematopoietic stimulating agents. Anlotinib, an oral targeted drug, was introduced from the second cycle onwards. Following completion of the sixth cycle, an abdominal CT scan revealed a reduction in tumor size from 4.8cm*4.0cm to 4.2cm*2.8cm in the region anterior to the aorta and postcava region. Subsequently, the patient underwent another surgical procedure.

**Table 1:** Details of chemotherapy regimen and drug usage and dosage.

Chemotherapy regimen (Chemotherapy cycle)	Drugs	does	Administration route	Days of administration
CAV (1,3,5,7,9)	vincristine	1.5mg/m^2^	i.v.	Day 1
	pirarubicin	50mg/m^2^	i.v.	Day 1
	cyclophosphamide	1.0g/m^2^	i.v	Day 1
VIP (2,4,6,8,10)	isocyclophosphamide	1.8g/m^2^	i.v.	Day 1–4
	etoposide	100mg/m^2^	i.v.	Day 1–4
	cisplatin	25mg/m^2^	i.v.	Day 1–4
VIT (11–16)	vincristine	1.5mg/m^2^	i.v	Day 1
	irinotecan	20mg/m^2^	i.v	Day 1–5; Day 8–12
	temozolomide	100mg/m^2^	p.o. (1 hour before intravenous infusion of irinotecan)	Day 1–5

#### Second stage surgery

In the second- stage operation, the tumor in front of the aorta and postcava, the greater omentum, the retroperitoneal lymph nodes and the hepatic hilum lymph nodes, and the visible tumors in the abdomen were removed.

After the second- stage surgery, CAV-VIP alternating chemotherapy was continued. At the end of the 4^th^ cycle post operation, radiotherapy was started. A total of 25 times of radiotherapy were delivered to abdominal cavity, pelvic cavity and pelvis, with a single fraction of 180gy and a total dose of 4500 Gy. Considering that bone marrow suppression was severe, radiotherapy was followed by VIT chemotherapy regimen which comprises vincristine, irinotecan and temozolomide for 6 cycles (**Table 1**).

#### Postoperative follow-up

An abdominal CT scan conducted 11 months after second-stage surgery did not reveal any recurrence of abdominal tumors (**Figure 1G**). However, bone metastases persisted. The patient is currently receiving oral targeted therapy with anlotinib while ongoing follow-up continues.

## Discussion

Desmoplastic small round cell tumor (DSRCT) is a rare aggressive tumor that predominantly arises from the peritoneum or retroperitoneum. It can invade the omentum through multiple peritoneal implants and metastasize to other organs, such as the diaphragm, splenic hilum, small intestine and large intestine mesentery, and pelvic peritoneum (5–7). Patients may be asymptomatic for months or even years, until they develop symptoms such as pain, ascites, constipation, weight loss, abdominal distension and jaundice (5, 6). This was the case for this patient presented in this report.

Because renal-derived DSRCT is rare, and DSRCT has no specific imaging features, the diagnosis of this disease was not considered prior to surgery. In retrospect, the tumor in this case did not break through the affected kidney, but had already metastasized to distant locations. This may be one of the differences between this disease and other renal malignant tumors. For instance, nephroblastoma, a common type of kidney cancer, rarely metastasizes to distant sites before breaking through the renal capsule. About one-half of the patients will present extra-peritoneal metastasis at the time of diagnosis (1, 6–9) and the liver and lung are the two most common sites for distant metastasis (8, 10). The absence of liver and lung metastases was observed in this case. However, it is noteworthy that bone metastases did manifest in the patient. Firstly, based on multiple imaging examinations and the interpretations provided by several radiologists, the lesion detected on the bone was deemed to be a metastatic tumor. Secondly, we conducted a comprehensive examination which included bone marrow aspiration and pathological analysis of all specimens obtained during both two surgeries; no additional tumors were identified. Thirdly, literature reports have documented instances of this tumor being found in bones as well (9, 10). In conclusion, a diagnosis of DSRCT involving the left kidney with abdominal lymph node metastasis and systemic bone metastasis has been established.

In our subsequent whole-genome NGS (next-generation sequencing technology) testing, we found that the child had a copy number amplification of the IL-7R gene in the NGS test results, which has a copy number of 3.5 in plasma. This gene mutation may regulate the overexpression and phosphorylation of c-fos and c-Jun, promote the formation of c-fos and c-Jun dimers, and then regulate the transcription and expression of the VEGFR-D gene. This promotes the process of cell malignant transformation, invasion, formation of tumor lymphatic vessels, and metastasis, and then participates in the occurrence and development of tumors (11).

Based on the previous knowledge and experience of renal malignant tumors, surgery is not recommended when distant metastases have occurred. In this case, the patient had already exhibited signs of cachexia and poor general condition before surgery. Therefore, the advice of multiple pediatric urology experts and pediatric oncology experts was to take a lymph node biopsy or tumor puncture biopsy to clarify the pathological diagnosis before undergoing chemotherapy or radiotherapy.

The patient’s preoperative imaging showed that the primary tumor site in the left kidney had an intact renal capsule and was still demarcated from the surrounding tissues, making it amenable to surgical resection. We believe that even if the tumor cannot be completely removed, removing the primary tumor could help to reduce the tumor burden in the patient’s body and prolong survival. After detailed communication with the patient’s parents, they accepted our treatment plan, and we performed a one-stage surgery for him.

Although we used distilled water to soak the tumor bed during the operation, the child inevitably developed peritoneal metastasis after the operation. It may be necessary to further explore what measures can be taken during the operation to minimize tumor implantation as much as possible.

The child obtained a definitive diagnosis based on pathological examination after the first operation, and the overall nutritional status improved, with the patient gaining significant weight, achieving good results. Therefore, we believe that the decision to operate on this patient was correct. Although the one-stage operation could not completely clear the tumor, reducing the tumor burden indeed help to improve the patient’s quality of life and prolong survival. In addition, reducing the tumor burden in the body may have a positive effect on the efficacy of subsequent chemotherapy.

Therefore, for the treatment of this type of malignant tumor with distant metastasis, we need to discuss whether we should not be passive in treatment. Especially when the tumor pathological type cannot be clarified, if the tumor can still be resected based on imaging findings, should we still be active in surgical resection?

Current treatment options for DSRCT include chemotherapy, radiation therapy, and cytoreductive surgery with hyperthermic intraperitoneal chemotherapy (HIPEC) (9, 12, 13). Surgical resection is the primary goal, but there is no definitive effective chemotherapy regimen. We performed MSI testing and TMB testing on the patient’s tumor sample. MSI-H was not detected in the sample, and TMB was 1.1muts/mb, suggesting that DSRCT patients may not benefit from immunotherapy. Therefore, the patient did not receive immunotherapy.

There is currently no standard chemotherapy regimen for DSRCT. Although the CAV-VIP alternate regimen did not eradicate the metastatic tumor in the bone, it resulted in a reduction in the volume of the residual tumor in the abdominal cavity, and the patient was free of tumor recurrence in the abdominal cavity 11 months after the second surgery. Therefore, this chemotherapy regimen is at least partially effective and can be used as a reference for DSRCT.

Although overall survival remains poor, radiation therapy following surgery seems to improve outcome (14). This patient was received a total of 25 times of radiotherapy in abdominal cavity, pelvic cavity and pelvis. Therefore, local radiotherapy may also be effective in delaying tumor recurrence.

## Conclusion

In summary, DSRCT is an extremely rare and highly malignant tumor. Primary renal DSRCT is even rarer. Currently, there is no definitive effective chemotherapy regimen. Surgical resection of as much tumor as possible is an effective treatment for prolonging life expectancy. If the tumor cannot be completely removed in a single stage, staged surgery may be adopted. CAV-VIP alternating chemotherapy may be an effective treatment for patients with DSRCT. Chemotherapy combined with anti-angiogenic targeted drugs may also be effective for DSRCT.

## Data availability statement

The original contributions presented in the study are included in the article/supplementary material. Further inquiries can be directed to the corresponding author.

## Ethics statement

The studies involving humans were approved by Ethics committee of Xiamen University. The studies were conducted in accordance with the local legislation and institutional requirements. Written informed consent for participation in this study was provided by the participants’ legal guardians/next of kin. Written informed consent was obtained from the minor(s)’ legal guardian/next of kin for the publication of any potentially identifiable images or data included in this article.

## Author contributions

GH: Writing – original draft, Data curation. WH: Project administration, Writing – review & editing. ML: Data curation, Investigation, Writing – review & editing.
